# Reduced susceptibility to carbapenems in *Enterobacteriaceae* and antimicrobial resistance profile of *Escherichia coli* strains isolated from clinical and zoonotic sources in the Bamenda Municipality, Cameroon

**DOI:** 10.11604/pamj.2023.44.90.31326

**Published:** 2023-02-16

**Authors:** Marie Ebob Agbortabot Bissong, Kingsley Ngah Mobey, Vernon Muma, Philip Bainmbo Mkong

**Affiliations:** 1 Department of Biomedical Sciences, University of Bamenda, P.O. Box 39, Bambili, Cameroon,; 2 Department of Medical Laboratory Science, University of Bamenda, P.O. Box 39, Bamenda, Cameroon

**Keywords:** Antimicrobial susceptibility, carbapenem-resistance, *Enterobacteriaceae*, multi-drug resistance, *Escherichia coli*, clinical isolates, zoonotic isolates

## Abstract

**Introduction:**

food-producing animals harbour pathogenic and antibiotic resistant bacteria which can be transmitted to humans. Resistance to carbapenems may complicate treatment resulting to debilitating consequences. This study aimed at determining the susceptibility of Enterobacteriaceae to carbapenems and to compare the resistant patterns of E. coli strains isolated from clinical and zoonotic sources.

**Methods:**

this was a cross-sectional study involving patients presenting at the Bamenda Regional Hospital and abattoir samples. Clinical samples (faeces and urine) and zoonotic samples (cattle faeces) were cultured and isolates identified using API-20E. Enterobacteriaceae isolates were tested for their susceptibility to Carbapenems. The susceptibility of E. coli was tested against eight antibiotics on Mueller Hinton agar. Data was analysed using SPSS version 20.

**Results:**

Enterobacteriaceae isolates from clinical specimen showed susceptibility of 93.3% to carbapenems. Out of 208 isolates 14 (6.7%) were Carbapenem-resistant Enterobacteriaceae (CRE) while 30 (14.4%) showed intermediate resistance and 164 (78.9%) were susceptible. The predominant CRE were Proteus (7/16, 43.8%), Providencia (3/15, 20.0%) and E. coli (4/60, 6.7%) with E. coli being the most clinically significant CRE. Multiple drug resistance (MDR) was observed in 83% of E. coli isolates, with the highest resistance being against vancomycin (90, 81.8%), azithromycin (69, 62.7%) and doxycycline (68, 61.8%). Clinical isolates were significantly (P<0.05) more resistant to azithromycin, trimethoprim-suphamethoxazole and gentamicin than zoonotic isolates.

**Conclusion:**

CRE were detected among isolates and a high rate of multiple drug resistance was observed among E. coli isolates. Proper antibiotic policies and good hygiene/sanitation measures may curb the development/spread of CRE and MDR E. coli.

## Introduction

Members of the family Enterobacteriaceae are Gram negative facultative anaerobes which are natural inhabitants of the intestinal tract of humans and animals [1,2]. They ferment a wide range of carbohydrates, possess a complex antigenic structure, and produce a variety of toxins and other virulence factors. This family constitutes over 40 genera and 150 species with the most common genera being *Enterobacter, Escherichia, Klebsiella, Proteus, Providencia, Salmonella, Serratia*, and *Shigella* [1]. Most of these bacteria are harmless; however, they can cause serious opportunistic infections in humans. Different members of Enterobacteriaceae are known to cause different diseases ranging from intraintestinal manifestations such as diarrhea to extra-intestinal diseases including wound infections, pneumonia, septicemia, bacteremia, and meningitis [3]. The emergence of antimicrobial resistance among *Enterobacteriaceae* has been increasingly reported worldwide and has become a major threat to the provision of healthcare [4]. Carbapenem-resistant *Enterobacteriaceae* (CRE) are Gram-negative bacteria that are resistant to the carbapenem class of antibiotics [5].

Carbapenems are a class of broad spectrum beta-lactam antibiotic reserved as last line of therapy for severe infections caused by multidrug-resistant (MDR) Gram-negative bacteria [6]. This class includes antibiotics such as imipenem, meropenem, ertapenem and doripenem. Carbapenem resistance is considered as one of the major health problems worldwide especially as this limits the choice of selected antibiotics therapies to treat bacterial infections. The mechanism of resistance to carbapenem is varied and may include the production of carbapenemase that breakdown carbapenem, production of extended-spectrum beta-lactamase (ESBLs), or extended-spectrum cephalosporinase (Ampc) [7]. The production of carbapenemase is the main mechanism of resistance for Carbapenemase producing *Enterobacteriaceae*(CP-CPE). Carbapenemase was classified molecularly to three classes (A, B, and D). Although there are several types of carbapenemases, the *K. pneumoniae carbapenemase*(KPC), oxacillinase48 (OXA48) and the New Delhi metallo betalatamase (NDM-1) are the most common carbapenemases produced by *Enterobacteriaceae* family [4,8,9]. These enzymes confer resistance to virtually all beta-lactam agents, including penicillins, cephalosporins and monobactams. On the other hand, non-carbapenemase carbapenem resistance is mediated by a combination of mechanisms, notably the production of ESBL or AmpC in addition porin mutations [5,7]. CRE infections are usually associated with health care and include urinary tract infections, central line-associated bloodstream infections, medical device infections, wound infections, and pneumonia.[10]. Detection of CRE can be performed phenotypically by isolating the bacteria and performing the traditional antimicrobial susceptibility testing (AST) [5,8,9]. Other phenotypic tests for carbapenemase production include Metallo-B-lactamase test, modified Hodge test (MHT), CarbaNP, Carbapenem Inactivation Method (CIM) or modified CIM (mCIM). Meanwhile, molecular techniques can be used to detect carbapenemase genes. The global rise of carbapenem-resistant *Enterobacteriaceae* (CRE) is alarming and represents an increasing threat to healthcare delivery and patient safety [11]. In Africa the prevalence of CRE varies, ranging from about 2% to 60% [10]. CRE infections are usually associated with health care with relatively higher healthcare costs, prolonged hospital stays, treatment failures and mortality [11]. Colonisation of the digestive tract with CRE has been associated with high rates (up to 89%) of subsequent infection, most frequently pneumonia, followed by urinary tract infections, primary bloodstream infections, skin and soft tissue infections, and surgical site infections [12]. Eradication of CRE from the intestinal flora is difficult and has been attempted with oral, non-absorbable antibiotic treatment. However, the success of this approach has been limited by a number of factors such as; relapse, development of antibiotic resistance during treatment, and patient refusal [13]. Consequently, the rapid emergence and expansion of carbapenem-resistant gram-negative bacteria (CR-GNB) is an urgent global public health threat. Moreover, little is known about the geographical distribution of these strains of *Enterobacteriaceae* especially in developing countries which lack adequate surveillance systems to monitor and control the spread of resistance strains. Hence, this study which was designed to determine the occurrence of CRE in such resource limited set-ups and to provide preliminary data on CRE in Cameroon is of utmost importance.

*E. coli* and other members of the family *Enterobacteriaceae* are often the most common gram negative bacteria isolates in clinical laboratories and *E. coli* are among the predominant microbes in the gastrointestinal tract (GIT) of humans and other animals [1,2]. Although they are mostly commensals in the GIT, some strains are pathogenic and can cause severe infections; typically, the shiga toxin producing *E. coli* (STEC) present with severe symptoms including abdominal cramps, bloody diarrhea and vomiting, resulting in conditions such as Hemolytic Uremic Syndrome (HUS) and Hemorrhagic Colitis (HC) [14]. *E. coli* is the major cause of septicaemia, urinary tract infections and gastrointestinal disorder, and they are responsible for a wide variety of hospital and community-onset infections [15]. Diarrheal diseases caused by these organisms, especially in children, is a major public health problem in developing countries [16]. Commensal *E. coli* are known to play a significant role in the emergence and spread of antimicrobial resistance in pathogens. It has been suggested that fecal microbiota could be a primary source of *E. coli* causing urinary tract infections (UTIs) especially as these microbes can easily be transmitted via the fecal-perineal-urethral route [17,18]. However, a recent study reported a significant difference between isolates from faeces and urine by pulsed-field gel electrophoresis (PFGE) analysis [19]. Cattle are usually considered the main reservoir for *E. coli* and animal products such as beef are easily contaminated during slaughter and processing [20]. The role of animals in the spread of *E. coli* is evidenced by several reports that revealed zoonotic strains in food, environment and humans [21-25]. The high probability of exchange of genetic materials could propagate negative attributes such as virulence and antibiotic resistance among these commensals. Consequently, it is important to constantly monitor the occurrence of these pathogens in animals. Also, the fact that *E. coli* are opportunistic pathogens is a cause for concern especially as cross-contamination between human body parts may lead to diverse opportunistic infections. The human urinary tract (especially in females) is prone to faecal contamination due to its proximity to the anal region. It is against this backdrop that the present study was initiated to compare *E. coli* strains from human faeces, human urine and cattle faeces based on their antimicrobial susceptibility patterns.

Antimicrobial resistance (AMR) has become a serious global public health challenge. Despite several efforts put in place to curb the development and spread of AMR, there seems to be a progressive rise in AMR worldwide [23]. The extensive use of antimicrobial agents to improve human and animal health and agricultural productivity worldwide has contributed enormously to the development of resistance which presents a serious public health challenge [26,27]. In addition, high resistance rates have been described in bacteria isolated from food-producing animals [28]. This poses a significant public health concern especially as resistant strains can be transmitted from animals to humans [29]. As a result, worldwide surveillance is a necessary tool for global AMR response. WHO recommends countries to develop a national AMR action plan and to re-inforce surveillance systems in order to obtain standardized data on AMR for policy implementation [28]. However, most sub-Saharan African countries lack a national surveillance system that routinely generates representative data on antimicrobial use and resistance [28,30]. The emergence and spread of antibiotic resistance in *E. coli* is one of the few evolution processes that demand experimental studies. The resistance patterns of bacteria from clinical sources has been extensively studied [23,31-33]. However, reports on antimicrobial resistance of *Escherichia coli* from environmental/zoonotic sources in Cameroon are few [22,34] and there is need for the assessment of these susceptibility patterns for epidemiological purposes. The present study has as objectives, to determine the susceptibility of *Enterobacteriaceae* to cabapenems and comparing the resistance profile of *E. coli* isolated from human and zoonotic sources. Findings from this study will increase awareness on the importance of cattle in the transmission of resistance strains to humans as well as the need to implement strategies to prevent cross contamination with these organisms.

## Methods

**Study site**: his study was carried out in the Bamenda Municipality in Mezam Division of the North West Region of Cameroon. The Bamenda Municipality comprises of three sub-municipalities; namely, Bamenda I, II and II. Clinical samples were collected from patients presenting for consultations at the Bamenda Regional Hospital (BRH) located at Mankon in the Bamenda II Municipality. BRH is the major government hospital in the NWR that serves the Bamenda municipality and its environs. Zoonotic samples comprising of cattle faeces were collected from the Bamenda Municipal Abattoir located at Nkwen in the Bamenda III Municipality. All samples were analysed in the microbiology Unit of the BRH laboratory.

**Study design**: this was a cross-sectional study involving patients presenting with symptoms of urinary tract infections at the Regional Hospital Bamenda. Enrolment of study participants was done by convenient sampling while collection of zoonotic samples at the abattoir was done randomly.

**Ethical consideration**: ethical clearance for this study was obtained from the University of Bamenda Ethical Review Board *(Reference Number: 2019/080H/UBa/IRB)*. Administrative authorization was obtained from the Regional Delegation for Livestock and Animal Husbandry, North West Region for the collection and analyses of abattoir samples. Each participant´s consent was obtained prior to enrolment into the study.

**Sample collection**: clinical samples (faeces and urine) were collected into clean collection containers accordance to standard guidelines [35]. Midstream urine samples were collected into sterile wide neck urine containers for analysis. Approximately 100 grams of zoonotic samples (cattle faeces) were collected in wide-mouth containers. All samples were transported on ice to the laboratory for analysis.

**Isolation and identification of bacterial isolates**: bacterial isolation was done on two types of microbiological media (Eosine methylene blue (EMB) agar and Cystine-lactose-electrolyte-deficient (CLED) agar) using the streak plate technique. All culture media were purchased from Liofilchem, Roseto, Italy. Approximately 1g of faecal sample was emulsified in about 10mL of sterile normal saline and the suspension was inoculated on EMB agar. Meanwhile, a 10µl wire loop was used to inoculate urine samples unto CLED agar. The plates were incubated at 37°C for 24h. From both culture plates, characteristic cololonies were gram stained and all gram negative bacilli were kept for further identification. The Analytical Profile Index - *Enterobacteriaceae* (API-20E) test strips (BioMerieux, Marcy-l´Etoile, France) were used to distinguish between members of the family *Enterobacteriaceae* and the procedure was conducted as described by the manufacturer.

**Detection of carbapenem resistance among *Enterobacteriaceae* isolates**: to determine resistance to carbapenem, all *Enterobacteriaceae* isolates were subjected to antibiotic susceptibility testing against two carbapenem antibiotics: imipenem (10 µg) and meropenem (10 µg) (Liofilchem, Roseto, Italy). The Kirby-Bauer disk diffusion method was used to test the isolates as previously reported [35,36]. Briefly, standardized bacterial suspension of each isolate was spread on Mueller Hinton agar (MHA) (Liofilchem, Roseto, Italy) after which the antibiotic disks were placed on the media. The plates were incubated at 35°C for 18-20 hours. The CLSI breakpoints [36] were used to interpret the results and isolates were classified as sensitive, intermediate resistant or resistant. CRE was determined if an isolate had a zone of inhibition of ≤ 19mm to either imipenem or meropenem or to both antibiotics [36].

**Phenotypic Carbapenemase production in *Enterobacteriaceae* isolates**: all CRE isolates were tested for carbapenemase production using the modified Hodge test as previously reported [36]. A standardized suspension of *E. coli* ATCC 25922 was inoculated on MHA and allowed to dry. A meropenem disk (10 µg) was placed at the center of each plate and the test organism (overnight cultures) was streaked in a straight line from the edge of the disk. Three test isolates were inoculated per plate (90mm) and after incubation at 35°C for 18-20 hours, the MHA plate was examined for enhanced growth around the streaked test organism. Antibiotics susceptibility testing of *E. coli* isolates: all (110) isolates confirmed as *E. coli* (30 from human faeces, 30 from human urine and 50 from cattle faeces) using the API-20E test were tested for their susceptibility to 8 selected antimicrobials, namely: cefixime (5 µg), doxycycline (30 µg), gentamicin (10 µg), nitrofurantoin (100 µg), ciprofloxacin (30 µg), Co-trimoxazole (25 µg), azithromycin (15 µg) and vancomycin (30 µg). All antibiotic disks were obtained from Liofilchem, Roseto, Italy and the Kirby-Bauer disk diffusion method was used as previously reported [35,36].

**Data Analysis**: data collected were analysed using the IBM Statistical Package for Social Sciences (SPSS; version 20.0). The differences in proportions of categorical variables and statistical significance were assessed using the Chi-square test and a p-value less than 0.05 was considered statistically significant. Charts and tables were used to display the results.

## Results

**Distribution of *Enterobacteriaceae* isolates in human faecal and urine samples**: a total of 100 enterobacteriaceae isolates belonging to 10 genera were detected from faecal samples while 108 isolates belonging to 6 genera were detected in urine. The predominant bacteria isolated from urine were *Klebsiella pneumonia* (36, 33.3%) and *E. coli* (30, 27.7%) while *E. coli* (30, 30%), *Hafnia* (16, 16%) and *Providencia spp* (15, 15%) were commonly isolated from faeces. The distribution of isolates between sample type was not statistically significant (P>0.05).

**Susceptibility profile of *Enterobacteriaceae* to carbapenems (imipenem and meropenem)**: *Enterobacteriaceae* strains were isolated from clinical samples (faeces) and the susceptibility patterns are recorded in Table 1 and Table 2. Out of 208 isolates 14 (6.7%) were CRE while 30 (14.4%) showed intermediate resistance and 164 (78.9%) were susceptible. The CRE detected in this study were *Proteus* (7/16, 43.8%), *Providencia* (3/15, 20.0%) and *E. coli* (4/60, 6.7%). Generally, *Enterobacteriaceae* isolates showed reduced susceptibility of 93.3% to carbapenems (89.9% and 88.9% for imipenem and meropenem, respectively. All isolates from urine were susceptible to imipenem (Table 1) while only 79 out of 100 (79%) faecal isolates were susceptible to imipenem (Table 2). On the other hand, similar susceptibility rates (92% and 93%) against meropenem were observed among urine and faecal isolates, respectively. Among isolates from faeces, only *E. coli* and *Providencia spp* showed resistance (7%) to imipenem; meanwhile, the lone species with resistance (87.5%) against meropenem was *Proteus spp* (Table 2). Although no resistance was observed among isolates from urine, a relatively high rate (14.8%) of intermediate resistance against meropenem was recorded among urine isolates, involving all species except *Proteus* (Table 1).

**Table 1:** susceptibility of *Enterobacteriaceae* isolates from urine against imipenem and meropenem

Bacterial Isolate		Antibiotics n(%)				
	Imipenem			Meropenem		
	R	I	S	R	I	S
***Klebsiella pneumoniae*(n=36)**	0 (0.0)	0 (0.0)	36 (100)	0 (0.0)	6 (16.6)	30 (83.3)
***E. coli* (n=30)**	0(0.0)	0(0.0)	30(100)	0(0.0)	4(13.3)	26(86.7)
***Klebsiella spp***>**(n=15)**	0 (0.0)	0 (0.0)	15 (100)	0 (0.0)	4 (26.7)	11 (73.3)
***Salmonella* *spp***> **(n=12)**	0 (0.0)	0 (0.0)	12 (100)	0(0.0)	1(8.3)	11(91.6)
***Proteus*(n=8)**	0 (0.0)	0(0.0)	8(100)	0(0.0)	0(0.0)	8(100)
***Enterobacter* *spp* (n=7)**	0 (0.0)	0 (0.0)	7 (100)	0 (0.0)	1 (14.3)	6 (85.7)
**Total**	0 (0.0)	0(0.0)	108 (100)	0(0.0)	16(14.8)	92 (85.2)

R = Resistant; I = Intermediate resistance; S = Susceptible

**Table 2:** susceptibility of *Enterobacteriaceae* isolates from faeces against imipenem and meropenem

Bacterial Isolate	Antibiotic n (%)					
	Imipenem			Meropenem		
	R	I	S	R	I	S
***E. coli*** (n=30)	4 (13.3)	12(40.0)	14(46.7)	0(0.0)	0(0.0)	30(100)
***Klebsiella pneumonia***(n=1)	0(0.0)	0(0.0)	1(100)	0(0.0)	0(0.0)	1(100)
***Enterobacter spp*** (n=14)	0(0.0)	0(0.0)	14(100)	0(0.0)	0(0.0)	14(100)
***Citrobacter spp*** (n=4)	0 (0.0)	0 (0.0)	4 (100)	0(0.0)	0(0.0)	4(100)
***Salmonella spp*** (n=7)	0(0.0)	0(0.0)	7(100)	0(0.0)	0(0.0)	7(100)
***Proteus spp*** (n=8)	0(0.0)	0 (0.0)	8(100)	7(87.5)	0(0.0)	1(12.5)
***Edwardsiella spp*** (n=2)	0(0.0)	0 (0.0)	2 (100)	0 (0.0)	0 (0.0)	2 (100)
***Serratia spp*** (n=3)	0(0.0)	0 (0.0)	3(100)	0(0.0)	0(0.0)	3(100)
***Providencia spp*** (n=15)	3(20.0)	2(13.3)	10(66.7)	0 (0.0)	0 (0.0)	15 (100)
***Hafnia spp*** (n=16)	0 (0.0)	0 (0.0)	16 (100)	0 (0.0)	0 (0.0)	16 (100)
Total (n=100)	7 (7.0)	14 (14.0)	79(79.0)	7(7.0)	0 (0.0)	93(93.0)

R = Resistant; I = Intermediate resistance; S = Susceptible

**Phenotypic carbapenemase production**: none of the 14 *Enterobacteriaceae* isolates that were resistant to either imipenem or meropenem was positive for the MHT indicating that these CRE isolates were not carbapemase producers.

**Figure 1:**
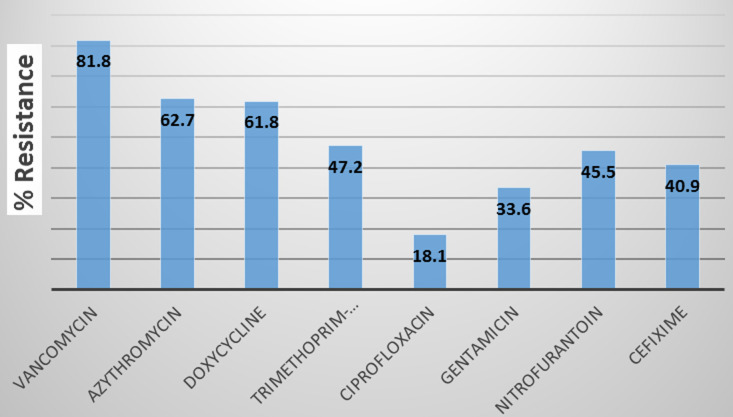
overall resistance of *E. coli* to different antibiotics

**Figure 2:**
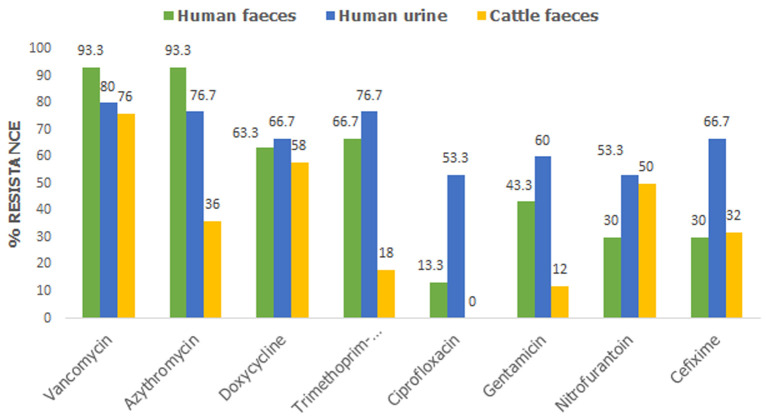
percentage resistance of the *E. coli* isolates from different sample types

**Antimicrobial resistance of *E. coli* isolates from clinical and zoonotic sources**: the resistance pattern of 110 *E. coli* isolates (30 from human faeces, 30 from human urine and 50 from cattle faeces) against a panel of 8 antibiotics was analysed and recorded in Figure 1. Generally, high resistance was observed against most of the antibiotics with vancomycin recording the highest (90, 81.8%) followed by azithromycin (69, 62.7%) then doxycycline (68, 61.8%). While ciprofloxacin (20, 18.1%) and gentamicin (37, 33.6%) recorded the lowest resistance. furthermore, the resistance patterns of isolates obtained from clinical and zoonotic sources were compared and the results are recorded in Table 3. Generally, clinical isolates were more resistant than zoonotic isolates and this difference was statistically significant (P<0.05) regarding azithromycin, trimethoprim-suphamethoxazole and gentamicin. In the same light considering all the sample types, isolates from human urine samples showed higher resistance than those from human and animal faecal samples (Figure 2). Additionally, urine isolates showed resistance of 50% and above to all (8, 100%) antibiotics tested as opposed to human faecal isolates (4, 50%) and cattle faecal isolates (3, 37.5%). Meanwhile, no resistance was recorded against ciprofloxacin among cattle faecal isolates.

**Table 3:** antibiotic resistance of *E. coli* isolates from clinical and zoonotic sources

Antibiotic		% Resistance		P value
Class	Type	Clinical Samples n (%) N=60	Zoonotic Samples n (%) N=50	
**Glycopeptides**	Vancomycin	52 (86.7)	38(76.0)	0.129
**Macrolides**	Azythromycin	51(85.0)	18(36.0)	0.023
**Tetracyclines**	Doxycycline	39(65.0)	29(58.0)	0.712
**Sulphonamides**	Trimethoprim-Sulphamethoxazole	43 (71.7)	9(18.0)	0.000
**Quinolones**	Ciprofloxacin	20 (33.3)	0 (0.0)	-
**Aminoglycosides**	Gentamicin	31(51.7)	6(12.0)	0.033
**Nitrofurans**	Nitrofurantoin	25(41.7)	25(50.0)	0.056
**Cephalosporins**	Cefixime	29(48.3)	16 (32.0)	0.462

**Figure 3:**
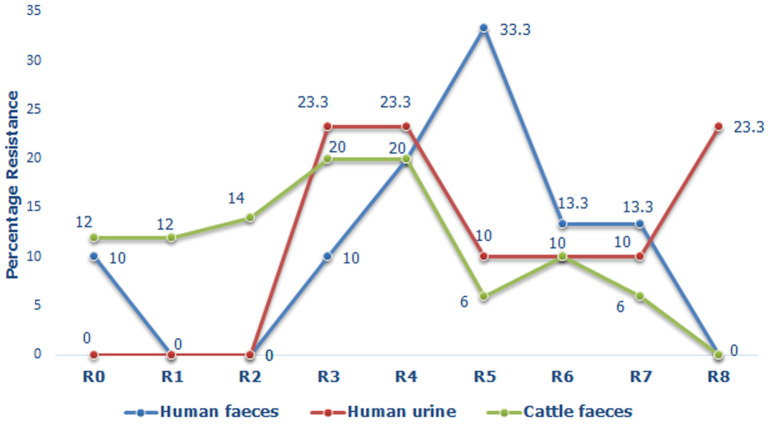
trends in the resistance type of MDR *E. coli* from different samples

**Multidrug-resistant (MDR) *E. coli* isolates**: Figure 3 shows the distribution of different resistance types of MDR *E. coli* isolated from various samples. The resistance types were categorized from R0 to R8 based on the number of antibiotic the isolate was resistant to. R0 showed no resistance to any of the antibiotics while R1, R2, R3, R4, R5, R6, R7 and R8 were resistant to 1, 2, 3, 4, 5, 6, 7, and 8 antibiotics; respectively. The isolates were considered multidrug-resistant if they were resistant to any three or more antibiotics (In this study, R3 through R8 were MDR). Generally, isolates from all sample types revealed a unique trend of resistance which peaks at R3, R4 or R5 (Figure 3). Although the highest peak was demonstrated by isolates from human faeces, most urine isolates fell among the higher categories of resistance (R3 to R8). Furthermore, the overall rate of multiple drug resistance in this study was 80.0% (88 out of 110). Interestingly, all urine isolates (30, 100%) were MDR and none of these isolates were in the R0 category. Meanwhile, 27/30 (90.0%) of human faecal isolates were MDR and only 3 (10%) isolates were in the R0 category. On the other hand, 31/50 (62.0%) of the cattle faecal isolates were MDR and up to 12.0 % of the isolates were in the R0 category. The phenotypic diversity of antimicrobial resistance of MDR clinical isolates was higher than that of zoonotic isolates with the former demonstrating 15 different combinations while the latter had 8 different combinations. The resistance pattern “Van-Azm-Cot-Dox-Gen” resistant to 5 antibiotics namely: vancomycin, azithromycin, trimethoprim-sulfamethoxazole, doxycycline and gentamicin were the predominant (10, 17.5%) antibiotype for clinical isolates. On the other hand, the most common (8, 38.1%) resistance pattern in zoonotic isolates was “Van-Dox-Nit”, resistant to vancomycin, doxycycline and nitrofurantoin (Table 4).

**Table 4:** multidrug-resistance pattern of *E. coli* isolates from clinical and zoonotic sources

MDR Clinical Isolates n=57		MDR Zoonotic Isolates n=31	
Resistance Pattern	Proportion n (%)	Resistance Pattern	Proportion n (%)
Van-Azm-Cot-Dox-Cpr-Gen-Nit-Cfx	7 (12.3)	Van-Azm-Cot-Dox-Gen-Nit-Cfx	3 (9.7)
Van-Azm-Cot-Dox-Cpr-Gen-Cfx	2 (3.5)	Van-Azm-Cot-Dox-Nit-Cfx	1(3.2)
Van-Azm-Cot-Dox-Gen-Nit-Cfx	3(5.3)	Van-Azm-Cot-Dox-Gen-Cfx	4(12.9)
Van-Azm-Cot-Cpr-Nit-Cfx	4(7.0)	Van-Azm-Cot-Dox-Nit	2(6.5)
Van-Azm-Cot-Dox-Cpr-Gen	3 (5.3)	Van-Azm-Dox-Nit	7 (33.3)
Van-Azm-Cot-Dox-Gen	10 (17.5)	Van-Azm-Dox-Cfx	3 (9.7)
Azm-Cot-Cpr-Nit-Cfx	3 (5.3)	Van-Dox-Nit	8(38.1)
Van-Azm-Cot-Dox	6(10.5)	Van-Dox-Cfx	1(3.2)
Van-Azm-Dox-Gen	2(3.5)	Van-Cot-Dox-Gen -Nit	1(3.2)
Cot-Cpr-Nit-Cfx	3(5.3)	Van-Azm-Dox	1 (3.2)
Van-Azm-Dox-Cpr-Gen-Nit-Cfx	2 (5.3)	NA	NA
Van-Azm-Cot-Cfx	2 (3.5)	NA	NA
Van-Azm-Nit	3 (5.3)	NA	NA
Cot-Gen-Cfx	3(5.3)	NA	NA
Van-Azm-Cot	4(7.0)	NA	NA

## Discussion

Antimicrobial resistance (AMR) has become a serious global public health challenge. Despite several efforts put in place to curb the development and spread of AMR, there seems to be a progressive rise in AMR worldwide [23]. As a result, worldwide surveillance is a necessary tool for global AMR response. Since most sub-Saharan African countries lack a national surveillance system to track the spread of AMR [30], it is imperative that studies on AMR in all sectors be enhanced in order to generate representative data on antimicrobial use and resistance. The impact of AMR on the health care sector cannot be overemphasized especially as the emergence and spread of resistant pathogenic strains has hindered the effectiveness of antibiotic therapy in many clinical conditions [37]. This has resulted in increased mortality, morbidity as well as higher socio-economic costs. High AMR rates have also been described in bacteria isolated from food-producing animals [26] and cross-species transmission of resistant bacteria or resistance genetic elements from animals or environment to humans is possible [29]. The emergence and spread of antibiotic resistance in *Escherichia coli* is a significant problem to human health and one of the few evolution processes that demand experimental studies. The present study was designed to determine the susceptibility of *Enterobacteriaceae* to carbapenems and to compare the resistant patterns of *Escherichia coli* strains isolated from clinical sample with those from zoonotic sources in order to give insight to the importance of food-producing animals in the dissemination of resistant strains.

Carbapenems are one of the major antibiotics reserved for the treatment of multidrug-resistant bacteria in health-care systems. As a result, the development of resistance to this antibiotic by pathogens may complicate the treatment of such diseases with debilitating consequences. The prevalence of CRE is increasing worldwide with the occurrence in Africa ranging from about 2% to 60% [10]; representing a serious threat to healthcare delivery and patient safety [11]. CRE most frequently colonizes the digestive tract and screening certain high-risk individuals for CRE colonization is a CDC-recommended intervention that can help stop the spread of CP-CRE. In this study, we screened 208 *Enterobacteriaceae* isolates (100 from faeces and 108 from urine) for their susceptibility to two carbapenems (imipenem and meropenem). A total of 14 (6.7%) CRE were detected in which the predominant CRE were *Proteus spp, Providencia spp* and *Escherichia coli* and out of these, none of the CRE was carbapenemase-producing. The prevalence of CRE in this study was lower than those reported in some previous studies; 22.4% in Uganda [38], 15.2% in Nigeria [39], and 37.9% in India [40]. However, similar findings have been reported in which Olowo-Okere recorded 6.5% resistance to carbapenems with the predominant CRE isolate being *Escherichia coli* [41]. The differences in the prevalence of CRE have been attributed to differences in geographical location, infection control in health-care settings or antibiotic policy [10]. In our context, the low prevalence of CRE could be due to the fact that carbapenems are rarely used in our hospitals. The fact that Proteus and *Providencia spp* are intrinsically more resistant to some carbapenems [42] makes *Escherichia coli* the most clinically significant CRE detected in our study. However, no carbapemases were detected among CRE isolates in our study, indicating that resistance to carbapenem in our local isolates may be as a result of mechanisms other than carbapenemase production such as the influence of efflux pump or deficiency of porin expression.

Among the two carbapenems tested, a higher susceptibility to imipenem (89.9%) than meropenem (88.9%) was observed among *Enterobacteriaceae* isolates in this study. This result is contrary to reports by Witkowska and colleagues which detected higher susceptibility to meropenem (93.4%) than imipenem (84.5%) [43]. This discrepancy may be influenced by the fact that majority of our CRE isolates belong to the genera *Proteus and Providencia* which demonstrate intrinsic resistance to some carbapenems [42]. Worthy of note is the fact that all CRE detected in this study were isolated from faeces. The detection of carbapenem resistance in commensals is of clinical significance especially as such strains may be capable of spreading resistance to pathogens [17]. Although urine isolates from this study did not demonstrate resistance to any of the carbapenems, a high rate of intermediate resistance (16, 14.8%) against meropenem was observed among these isolates. This is a clear indication that the rate of CRE among pathogenic bacteria is most likely to increase in the study area in future. Consequently, the implementation of proper antibiotic policies and effective infection control in our health-care systems will be of utmost importance in reducing the development and spread of CRE. Furthermore, the resistance pattern of all (60) *E. coli* isolates from this study were compared with *E. coli* (50) isolated from cattle faeces in order to gain insight on the possibility of cross-contamination and the importance of food animals in the spread of resistance strains to humans. The isolation rate of *E. coli* in this study was 53.64%, with 68.3% from environmental samples and 41.7% from clinical samples. Meanwhile, more *E. coli* were isolated from faeces (53.3%) than urine (30%). This is obvious as these organisms are commensals in the gastrointestinal of animals and humans. In a similar study by Kibret and Abera, *E. coli* was isolated from 14.2% of clinical samples in which the highest isolation rate (45.5%) was obtained from urine samples [44].

In this study, high level of resistance of *E. coli* isolates were reported against vancomycin, azithromycin and doxycycline. This corroborates previous findings [44,45]. Studies by Kibret and Abera revealed high resistance rates to erythromycin (89.4%), amoxicillin (86.0%) and tetracycline (72.6%) [44]. Tanih *et al*. also reported vancomycin resistance in all (100%) of *E. coli* isolated from cattle and pigs [45]. In a review describing the current state of AMR in Cameroon, it was revealed that *E. coli* strains from humans showed high resistance to trimethoprim-sulphamethoxazole (85.2%), tetracycline (71.9%), amoxicillin (77.3%), nitrofurantoin (71.9%) and doxycycline (45%) [46]. The high resistance (77.8%) of urinary *E. coli* to trimethoprim-sulphamethoxazole in this study is indicative that this drug may not be potent in treating UTI in the study area, contrary to CDC recommendation for the treatment of uncomplicated UTI with trimethoprim-sulfamethoxazole [47]. Meanwhile, ciprofloxacin (11, 18.6%) and gentamicin (20, 33.9%) recorded the lowest resistance in our study. This result is similar to previous reports [44,48]. In one of these studies Nzalie *et al*. reported low resistant rates of *E. coli* against intravenous drugs such as gentamicin and ceftriaxone as well as in oral fluoroquinolones from cases of community-acquired UTI. Also, low resistance of *E. coli* to gentamicin has been reported both in human and zoonotic sources [31,46]. Furthermore, the resistance patterns of isolates obtained from clinical and zoonotic sources were compared and it shows that clinical isolates were significantly more resistant to azithromycin, trimethoprim-suphamethoxazole and gentamicin than zoonotic isolates. It is obvious that macrolides and aminoglycosides are rarely used for livestock production in Cameroon, as a result, there is less exposure of zoonotic microbes and low resistance to these antibiotics. According to a recent study, antibiotics commonly used in poultry farms in Cameroon include fluoroquinolones, tetracyclines and sulphonamides [49]. But in our study, no resistance was recorded against ciprofloxacin among isolates from cattle faeces. This may be explained by the fact that cattle breeding in Cameroon is yet to be intensive and as a result, the use of antibiotics in this sector is limited. However, similar to our study, high resistance to quinolones in the clinical settings have been reported [32]. Considering all the sample types used in this study, isolates from urine showed higher resistance than those from human and animal faeces. In addition, urine isolates showed resistance of >50% to all (8, 100%) antibiotics tested as opposed to human faecal isolates (4, 50%) and cattle faecal isolates (3, 37.5%). This result is concurrent with previous findings in which Bahadora *et al*. reported high antimicrobial resistance among human urinary isolates than faecal isolates. In their study, PFGE patterns revealed a significant difference in *E. coli* from urine and faeces [19].

Generally, a high rate (83%) of multiple drug resistance was observed among *E. coli* isolates in this study. Similarly, high rates of MDR have previously been reported in the clinical setting as well as in zoonotic sources [19,25] and this has been attributed to increasing use and/or misuse of these agents in human health and animal production [29]. Contrary to our results, Li *et al*. reported low prevalence of MDR in pigs [50]. In addition, the resistant patterns were different in isolates from different sources. MDR *E. coli* were isolated more from human faeces (93.8%) than cattle faeces (73.5%) meanwhile, all urine isolates were MDR. This is suggestive that humans in the study area may be more exposed to antimicrobial use than livestock. Worthy of mention is the fact that all vancomycin-resistant *E. coli* isolates presented with multidrug-resistance. This study is one of a few studies that have evaluated the antimicrobial resistant profile of clinical and zoonotic isolates in the Bamenda Municipality and it provides baseline information on differences between commensal and pathogenic *E. coli*. Data processed in this study makes available valuable knowledge and information that could help in the prevention, prospects and management of infections caused by bacteria of the family *Enterobacteriaceae* in our localities. Findings of this study has restated the need for proper handling of food (especially beef), slaughter equipment and abattoir wares, and waste disposal (cattle faeces) as resistant *E. coli* isolates were found in both clinical and zoonotic samples.

**Limitations**: this study was limited by the following aspects: 1) molecular techniques which are more reliable in confirming the identity of isolates were not employed in this study; 2) resistance to carbapenems was tested only against clinical isolates; 3) the fact that none of the CRE isolates in this study were positive for carbapenemase production may be accounted by the relatively small number (14) of CRE tested. Subsequent studies are recommended to screen a larger sample size so as to obtain sufficient numbers of CRE for further characterization.

## Conclusion

*Enterobacteriaceae* isolates from clinical specimen showed reduced susceptibility of 89.9% and 88.9% to imipenem and meropenem, respectively. A total of 14 (6.7%) CRE were detected in which *E. coli* was the most clinically significant CRE. A high rate (83%) of multiple drug resistance was observed among *E. coli* isolates from clinical and zoonotic sources, with the highest resistance being against vancomycin, azithromycin and doxycycline. However, clinical isolates were significantly more resistant to azithromycin, trimethoprim-suphamethoxazole and gentamicin than zoonotic isolates. The high resistance (77.8%) of urinary *E. coli* to trimethoprim-sulphamethoxazole is a cause for concern that may necessitate modification in the treatment of uncomplicated UTI. The detection of CRE in human faecal samples and the high rate of intermediate resistance (16, 14.8%) among urinary pathogens may indicate a likely increase in the proportion of CRE among pathogenic bacteria in the study area. Consequently, the implementation of proper antibiotic policies, good hygiene/sanitation measures in the abattoirs and effective infection control in our health-care systems will help to reduce the development and spread of CRE and MDR *E. coli. Enterobacteriaceae* isolates from clinical specimen showed susceptibility of 93.3% to carbapenems. Out of 208 isolates 14 (6.7%) were Carbapenem-resistant *Enterobacteriaceae* (CRE) while 30 (14.4%) showed intermediate resistance and 164 (78.9%) were susceptible. The predominant CRE were Proteus (7/16, 43.8%), Providencia (3/15, 20.0%) and *E. coli* (4/60, 6.7%) with *E. coli* being the most clinically significant CRE. Multiple drug resistance (MDR) was observed in 83% of *E. coli* isolates, with the highest resistance being against vancomycin (90, 81.8%), azithromycin (69, 62.7%) and doxycycline (68, 61.8%). Clinical isolates were significantly (P<0.05) more resistant to azithromycin, trimethoprim-suphamethoxazole and gentamicin than zoonotic isolates. CRE were detected among isolates and a high rate of multiple drug resistance was observed among *E. coli* isolates.

### What is known about this topic

- *Prevalence of CRE in other regions of Cameroon;*
- *Antibiotic resistance of E. coli isolates from human sources;*
- *Antibiotic resistance of E. coli isolates from animal sources.*

### What this study adds

- *This study was the first to report carbapenem resistance among Enterobacteriaceae isolates in the North West Region of Cameroon with a prevalence of 6.7%;*
- *This study revealed that E. coli was the most common CRE of clinical significance in the study area;*
- *It was observed that clinical isolates of E. coli were more resistant to azithromycin, trimethoprim - suphamethoxazole and gentamicin than zoonotic isolates.*
